# Synaptic correlates of benzodiazepine tolerance

**DOI:** 10.1038/s41380-026-03512-8

**Published:** 2026-02-26

**Authors:** Alexandre Piot, Jasmina N. Jovanovic

**Affiliations:** UCL School of Pharmacy, University College London, London, WC1N 1AX UK

**Keywords:** Neuroscience, Diseases

## Abstract

Benzodiazepines are among the most widely prescribed psychotropic agents, exhibiting anxiolytic, hypnotic, anticonvulsant, and myorelaxant properties primarily mediated through positive allosteric modulation of type A γ-aminobutyric acid receptors (GABA_A_Rs)—the principal inhibitory receptors in the mammalian brain. However, prolonged benzodiazepine administration frequently results in the development of tolerance, a phenomenon characterized by reduced pharmacological efficacy and the need for escalating doses to sustain therapeutic benefit. Accumulating evidence indicates that benzodiazepine tolerance arises from complex neuroadaptive processes that induce long-lasting reorganization of neural circuits regulating stress responsiveness, arousal, and reward processing. In this review, we synthesize current knowledge on the clinical and societal impact of benzodiazepine use, delineate the molecular targets and mechanisms underlying their psychotropic effects on brain physiology and behaviour, and provide a comprehensive overview of the molecular and cellular adaptations contributing to benzodiazepine tolerance and withdrawal. Particular emphasis is placed on how prolonged exposure remodels inhibitory and excitatory synaptic organization. Furthermore, we examine evidence for benzodiazepine-induced synaptic adaptations in patients and highlight the potential of integrative in vivo imaging approaches to elucidate these processes within the complexity of the human brain. Finally, we propose that advancing these research avenues could foster a paradigm shift in the clinical use of benzodiazepines and guide the development of next-generation therapeutics with improved safety and efficacy profiles.

## Introduction

Benzodiazepines are potent psychotropic drugs that have been prescribed for treating stress-related disorders over many decades due to their rapid onset of action, potency and low toxicity. The prevalence of these conditions leads to an estimated several million prescriptions of benzodiazepines every year in the UK (UK Advisory Council on the Misuse of Drugs, 2020) [1]. In addition, benzodiazepines are widely used for nonmedical purposes having been listed among the common illegally supplied medicines in the UK [2]. In the United States, prescribing benzodiazepines in primary care is widespread with the number of prescriptions being as high as 65.9 million per year [3]. Moreover, data from the 2018 US National Survey on Drug Use and Health show that approximately 2% of the US population aged 12 or older reported misuse of benzodiazepines in 2017. Data also indicate that the number of deaths involving benzodiazepines increased by 43% from 2019 to 2020 in the US, 92.7% of which involved opioids, and most commonly illicit fentanyl [4]. This demonstrates that benzodiazepines alone rarely cause death but can be hazardous when combined with other CNS depressants such as opioids [5]. Globally, benzodiazepines are the most widely misused prescription drugs, and, in 2018, they were listed among the three most misused substances in as many as 60 countries (United Nations Office on Drugs and Crime. World Drug Report, 2018).

The initial highly beneficial, anti-convulsant, sedative-hypnotic, anxiolytic and muscle relaxant effects of benzodiazepines are over-time surpassed by the development of tolerance and dependence, resulting in severe withdrawal symptoms upon drug cessation. In many countries and the UK, clinical use of benzodiazepines is nowadays limited to treating severe, disabling anxiety, panic attacks, some forms of epilepsy or insomnia for maximum of 2–4 weeks. The recommended first line therapies for acute anxious symptoms are, in fact, slow acting antidepressants – selective serotonin or serotonin/noradrenaline reuptake inhibitors (SSRIs or SNRIs, respectively) – and anxiolytics such as gabapentinoids (gabapentin, pregabalin), although the clinical utility of these agents in anxiety and insomnia remains inconsistent and is often offset by sedation [6–9] and cognitive and respiratory adverse effects [10–12].

An estimated 35% of patients in the UK continue taking benzodiazepines for a significantly longer time [13], indicating that prescribing benzodiazepines in healthcare practice often exceeds the duration and dose recommended by the guidelines [1, 14]. This paradox can be explained at least in part by the fast and predictable initial symptom relieving effects of benzodiazepines, but also by the slow onset of the effects of the alternative drug treatments for anxiety and insomnia mentioned above.

Benzodiazepines are a class of over 30 chemically related psychoactive drugs that are often classified according to their half-life into three main categories: short (e.g. Lorazepam, Temazepam, Alprazolam; t_1/2_ = 2–15 h), intermediate (e.g. Flunitrazepam, Oxazepam; t_1/2_ = 15–30 h), and long (e.g. Diazepam, Clobazam; t_1/2_ = 30–50 h; Table 1) [15], although this classification is becoming increasingly confined with the development of new benzodiazepines such as remimazolam with an ultra-short action of about 30 minutes [16]. The archetypal structural component of benzodiazepines is a seven-membered ring to which an aromatic ring is bound [17]. Benzodiazepines are mainly ingested in the form of a pill, reaching a maximum concentration in plasma after a couple of hours and slowly declining thereafter [18]. Due to their high lipophilicity, benzodiazepines rapidly cross the blood–brain barrier, with brain concentrations closely tracking the unbound plasma fraction; protein binding and lipophilicity together determine their distribution and elimination [19, 20]. Given the lipophilicity and long half-life of certain active metabolites, benzodiazepines can accumulate in the body resulting in side effects such as lethargy, fatigue, impaired motor coordination, slurred speech, and confusion [19]. Tolerance to the effects of benzodiazepines develops at different rates. First, tolerance to the sedative effects of benzodiazepines develops, followed by tolerance to the anticonvulsant effects [21, 22]. By contrast, tolerance to the anxiolytic and amnestic effects of benzodiazepines has been shown to take a long time to develop in animal studies [21, 22] but has not been readily demonstrated in human patients [21] except for short-acting benzodiazepines [23, 24]. However, long-term treatment of anxiety disorders with benzodiazepines has been associated with an increased risk of developing substance use, mood disorders [25] and suicide [26].

**Table 1:** Pharmacological characteristics of selected benzodiazepines.

Compound	Parent half-life (hours)	Clinical dosage	Primary indication (UK)	Primary indication (US)	Equivalent to 10 mg Diazepam	Time to Tolerance development (effect/population)	Subunit selectivity	Active metabolites half-life (hours)
***Short acting (t***_***1/2***_ = ***2–15 h)***								
Temazepam	7–14	10–40 mg; 1x daily	Insomnia	Insomnia	20 mg	Lorazepam > Temazepam (sedative effects/humans)	α1, α2, α3 or α5	4–15
Alprazolam	12–15	0.25–3 mg; 3x daily	Anxiety	Anxiety	0.5 mg	Flunitrazepam > Alprazolam (hypnotic/insomniacs)	α1, α2, α3 or α5	1
Lorazepam	2	0.5–4 mg; divided daily	Anxiety	Anxiety	1 mg	Lorazepam > Temazepam (sedative effects/humans)	α1, α2, α3 or α5	—
Midazolam	1.5–2.5	2–10 mg	Epilepsy	Epilepsy	n/a	Flurazepam> Midazolam (hypnotic effects/insomniacs)	α1, α2, α3 or α5	0.8–1
***Intermediate acting (t***_***1/2***_ = ***15–30 h)***								
Flunitrazepam	10–30	0.5–1 mg; 1x daily	Not licensed	Not licensed	n/a	Flunitrazepam > Alprazolam (hypnotic/insomniacs)	α1, α2, α3 or α5	20–30
Oxazepam	30	10–30 mg; 3x daily	Anxiety	Anxiety	20 mg	Diazepam > Oxazepam (psychomotor and cognitive effects/ human with history of drug abuse)	α1, α2, α3 or α5	—
***Long acting (t***_***1/2***_ = ***30–50 h)***								
Diazepam	24–48	1–10 mg; 3x daily	Muscle spasm	Anxiety	—	Diazepam > Oxazepam (psychomotor and cognitive effects/ human with history of drug abuse)	α1, α2, α3 or α5	≤200
Clobazam	50	20–30 mg divided daily	Epilepsy	Lennox-Gastaut syndrome	20 mg	No tolerance to antiepileptic effects (anticonvulsant/humans)	α2 preferring	20
Flurazepam	2–3	15–30 mg 1x daily	Insomnia	Insomnia	15–30 mg	Flurazepam> Midazolam (hypnotic effects/insomniacs)	α1, α2, α3 or α5	≤100

Parent compound and active metabolite half-life data are from Soyka et al. [15]. Indicative clinical dosages refer to oral administration in adults for non-emergency conditions, as listed in the British National Formulary (BNF; https://bnf.nice.org.uk/). U.S. primary indications are taken from the FDA Medication Guide database (https://dps.fda.gov/medguide).The most widely prescribed benzodiazepines have comparable affinities and efficacies for GABA_A_R subtypes composed of α1βγ2, α2βγ2, α3βγ2, or α5βγ2 subunits and thus produce comparable behavioral effects as combined output of α₁ (sedation, amnesia), α₂/α₃ (anxiolysis, myorelaxation), and α₅ (memory modulation)[160].Comparative data on tolerance development are derived from studies that did not always employ equipotent dosing, and results should therefore be interpreted with caution.

In vivo pharmacodynamic potency and pharmacokinetic half-life differences between different types of benzodiazepines impact the rate and extent of tolerance development [27]. Tolerance can develop without symptoms of dependence and conversely, dependence can occur without signs of tolerance, and neither has been directly linked to fluctuations in serum or tissue concentration of benzodiazepines which are known to remain stable during chronic treatments in both animal models and patients [22, 28]. However, tolerance increases the risk of dependence and debilitating withdrawal symptoms. Benzodiazepine withdrawal is characterized by anxiety and anxiety-related symptoms (sleep disturbances, irritability, panic attacks, hyperventilation, tremor, visual disturbance, weight loss), perceptual changes, and in some cases seizures and precipitation of psychosis [29]. Thus, the symptoms of benzodiazepine withdrawal resemble the symptoms of disorders benzodiazepines attempt to treat, suggesting that benzodiazepine tolerance and withdrawal may be two manifestations of the same homeostatic neuronal mechanism [30]. Effective in reducing the severity of withdrawal symptoms is flumazenil (Ro 15–1788) [31], benzodiazepine antagonist, which is also used in acute clinical setting for benzodiazepine overdose [32]. However, rapid flumazenil administration can precipitate acute withdrawal, including agitation and seizures, and therefore requires careful monitoring, particularly in benzodiazepine dependent or tolerant patients [33]. Nonetheless, limited clinical evidence also suggests that low dose or slowly titrated flumazenil may be beneficial in the management of tolerance [34].

Advances in molecular characterisation of benzodiazepine targets in the central nervous system have laid the groundwork for extensive efforts in the pharmaceutical industry to generate new benzodiazepine compounds with fewer side effects and more specific clinical outcomes, however, with very limited success. Several new benzodiazepine-like compounds progressed into clinical development but were halted in clinical trials due to toxicity or the lack of specificity (recently reviewed in [35, 36]).

## Benzodiazepine targets in the CNS

The fundamental principle of benzodiazepine action in the brain is that they bind with high affinity to GABA_A_Rs, the primary inhibitory receptors in the brain. These ligands allosterically enhance the receptor’s responsiveness to GABA [27, 37–39], the principal inhibitory neurotransmitter in the mature central nervous system (CNS) [40]. In tandem with glutamate, the main excitatory neurotransmitter, GABA produces the precise homeostasis required for normal brain function [41]. Many psychiatric diseases including epilepsy [42, 43], anxiety [44], depression [45] and schizophrenia [46] have been associated with dysregulation of GABAergic transmission, underscoring its fundamental role in the brain.

GABA_A_Rs are ligand-gated chloride/bicarbonate channels built up as heteropentamers from a pool of 19 different subunits classified as α(1–6), β(1–3), γ(1–3), δ, ε, π, θ and ρ(1–3). The subunit composition determines their functional properties, pharmacological sensitivity, and regional expression [47–49]. By inducing a global conformational change in the receptor which increases its conductance without altering its apparent binding affinity for GABA [50], benzodiazepines potentiate chloride currents of GABA_A_Rs. This conformational change was estimated to increase the stability of the pre-activation stage of the receptor four-fold, possibly due to stabilization of the interactions between the subunits that form the benzodiazepine binding pocket (α and γ subunits; [51]). This has been proposed to facilitate channel opening upon GABA binding to two specific binding sites situated at the interface  between the α and β subunits causing a leftward shift in the concentration-response curve compared to that of GABA alone [50]. Benzodiazepines are strictly GABA-dependent positive allosteric modulators and cannot open GABA_A_ channels on their own [52] which may be a contributing factor to their higher therapeutic index compared to other GABA_A_R ligands, for example barbiturates. Barbiturates prolong GABA_A_ channel open time and, at higher concentrations, can directly gate the channel in the absence of GABA [53]. In addition, whereas benzodiazepines display functional selectivity based on subunit composition, barbiturates bind at the α-β subunit interface that is shared across most GABA_A_R receptor isoforms. Barbiturates therefore potentiate a wider range of receptor subtypes [54], leading to a high risk of profound CNS/respiratory depression.

The classical binding site of benzodiazepines lies at the interface between the α and γ subunits, but newer structural analyses have identified additional binding sites at the α-β interface, which can potentiate further receptor activation [55]. Whether and how these additional binding sites contribute to the development of benzodiazepine tolerance remains to be established.

Benzodiazepine binding differs at the α-γ site interface [56] imparting the selectivity of certain benzodiazepines for specific α subunits [57]. It is also well established that subunit composition of GABA_A_Rs determines the type of physiological responses to benzodiazepines treatments. The α_1_-GABA_A_Rs mediate the sedative, amnesic and anticonvulsant effects of diazepam [58], whereas the α_2_-GABA_A_Rs contribute to the anxiolytic and myorelaxant effects of benzodiazepines [59]. Myorelaxation is also mediated by the α_3_-GABA_A_Rs [60] and α_5_-GABA_A_Rs [61]. The amnesic effects of benzodiazepines have been predominantly attributed to α_5_-GABA_A_Rs, which are the most abundantly expressed in the hippocampus [62]. The variety of physiological effects mediated by the α subunits can be explained by their region-, tissue- and synapse-specific localization in the brain [63].

Benzodiazepine-sensitive GABA_A_Rs are predominantly located at GABAergic synapses formed by morphologically and functionally distinct types of inhibitory interneurons and their target cells. GABAergic synapses are essential for the fast transmission of inhibitory signals mediated by GABA [63]. They have a distinct morphological organisation which allows activity-dependent release of GABA from the presynaptic elements (also termed axon terminals; [64, 65]) and its fast diffusion across the synaptic cleft to reach GABA_A_Rs clustered at the postsynaptic membrane. GABAergic synapses incorporate a large number of pre- and post-synaptic proteins which are important for their structural organisation and function in addition to GABA_A_Rs [66–69].

Synaptic GABA_A_Rs containing α_1__-__3,_ β_2-3_ and γ_2_ subunits are functionally distinct from extrasynaptic receptors composed of α_4-6_, β_2-3_ and δ subunit-containing GABA_A_Rs [64]. The latter have significantly higher affinity for GABA and are predominantly located outside of synapses where they can be activated by low levels of ambient GABA to mediate tonic inhibition [64]. Given that at clinically relevant concentrations benzodiazepines predominantly allosterically potentiate the synaptic γ_2_-GABA_A_Rs [39], the short- and long-term physiological effects of these drugs should be considered principally in the context of functional, structural and adaptive changes in GABAergic synapses.

Interestingly, some benzodiazepines also bind to the evolutionary conserved 18 kDa translocator protein (TSPO) otherwise known as the peripheral benzodiazepine receptor [70]. Among these are diazepam, midazolam, lorazepam and Ro5-4864, but not clonazepam [71]. TSPO is located on the outer membrane of mitochondria where it regulates mitochondrial respiration, Ca^2+^ homeostasis, oxidative stress, cell proliferation and programmed cell death [72]. In the brain, it is one of the well-established neuroinflammatory markers utilized in diagnostic imaging [73] because its expression is triggered by neuroinflammation [74] and brain injury [75]. In contrast to these conditions, anxiety disorders are characterized by low levels of TSPO expression [76]. In agreement with human studies, genetic deletion of TSPO in animal models results in anxiogenic phenotype while pharmacological activation of TSPO with the specific activator XBD-173 (Emanupil) produces anxiolytic effects in wild type but not TSPO deficient mice, indicating that these effects are TSPO-dependent [77]. TSPO thus plays an important role in anxiety- and depression-related behaviours, as demonstrated in animal models and humans [78]. In neurons, TSPO activity can be linked indirectly to GABAergic transmission due to its role in mitochondrial synthesis of neurosteroids, another class of potent allosteric modulators of GABA_A_Rs [79, 80]. However, the essential role of TSPO in neurosteroid production remains controversial due to conflicting results obtained in early pharmacological studies and various TSPO knockout models [80]. It is of interest to note that TSPO expression in microglia and neurons appears to increase during the course of benzodiazepine treatments in animal models [81], which may impact the development of tolerance. This is supported further by early studies indicating that neurosteroids can prevent the development of tolerance associated with chronic diazepam or triazolam treatments in mice [82]. However, clinical evidence implicating a role of neurosteroids in the development or attenuation of tolerance in patients is lacking.

Here we provide an overview of synaptic processes occurring during chronic exposure to benzodiazepines that may correlate with the development of tolerance. We argue that future developments in their clinical use aiming to reduce or eliminate tolerance and dependence in patients depend critically on our understanding of how benzodiazepines impact the structural and functional integrity of inhibitory and excitatory synapses in the brain.

## Short-term effects of benzodiazepines on inhibitory synapses

Continuous exposure to benzodiazepines over a relatively short period of time (up to 24 h) leads to functional and structural alterations in synaptic GABA_A_Rs that could potentially trigger the development of tolerance.

### Pharmacological uncoupling between benzodiazepine and GABA binding

One of the earliest proposed mechanisms for benzodiazepine tolerance is pharmacological uncoupling—a functional decoupling between the benzodiazepine binding site and the GABA binding site on GABA_A_Rs [21]. This hypothesis emerged from findings that benzodiazepines with the highest tolerance liability also exhibit pronounced desensitization of GABA_A_Rs and reduced [³H]-flunitrazepam binding [83]. Several studies in neuronal cell culture reported similar observations but with more rapid time course (reviewed in [21, 27, 30]). Further insights into the time course of uncoupling were obtained using the heterologous expression of GABA_A_Rs in HEK293 cells, in which the time required for uncoupling was even shorter [84]. Subsequent in vivo experiments in rats reported a fast onset of uncoupling within 12 h, returning to normal levels within 24 h [85], suggesting that this is a fast, reversible process.

While the precise mechanisms underlying uncoupling remain unclear, recent investigations into the short-term effects of benzodiazepines described molecular and cellular processes that could be considered as potential contributors. These include a) receptor-intrinsic changes, such as changes in subunit composition or activation state [86], b) post-translational modifications, including phosphorylation/dephosphorylation of β and γ subunits [87, 88], c) changes in receptor interactions with trans-synaptic or postsynaptic proteins, including Shisa7, a protein that can enhance the effects of diazepam on the receptor gating [89], d) synaptic remodelling, such as lateral diffusion of GABA_A_Rs between synaptic and perisynaptic locations or relocation of benzodiazepine-insensitive receptor subtypes to synapses [90], e) changes in scaffolding proteins, including gephyrin [91, 92] or radixin [93] which anchor GABA_A_Rs at inhibitory synapses. The quick onset of uncoupling within a matter of hours [30] is consistent with the dynamic and reversible pattern of these changes. However, benzodiazepine tolerance does not occur within hours but rather over days to weeks. Additionally, this gradual process is difficult to reconcile with the rapid reversal of uncoupling by flumazenil both in vitro [94] and in vivo [95]. This suggests that uncoupling between the benzodiazepine and GABA active sites is solely a short-term effect of benzodiazepine administration and therefore unlikely the mechanism responsible for the development of benzodiazepine tolerance [21].

### GABA_A_Rs internalization and intracellular processing

Another short-term effect of benzodiazepines is the reduction of GABA_A_R surface expression which results in accumulation of intracellular receptor pools that could be targeted for protein degradation or re-insertion into the synapses [96]. Multiple mechanisms could be involved in such processes including expression or synaptic localization of GABA_A_R-associated proteins, for example, gephyrin [97]. Interestingly, it has been proposed that internalization of GABA_A_Rs may be a subunit-specific effect of short-term exposure to specific benzodiazepines [98], which can be quickly reversed by application of flumazenil. However, these dynamic and reversible synaptic changes may or may not be long-lasting and thus their contribution to the development of tolerance remains in question. Nonetheless, prolonged exposure (beyond 24 h) shifts the balance from reversible receptor trafficking towards more sustained depletion of GABA_A_Rs [99] that may be at play during the early stages of benzodiazepine tolerance. This may involve changes in intracellular processing, including deficits in recycling of internalised GABA_A_Rs [100] and/or targeted degradation [101]. The reported increase in the proportion of endocytosed GABA_A_Rs present on clathrin-coated vesicles isolated from mice treated with lorazepam for a week [102] further supports this hypothesis.

## Long-term effects of benzodiazepines on inhibitory synapses

Continuous exposure to benzodiazepines over days or weeks leads to long-term changes in GABA_A_Rs and inhibitory GABAergic synapses that may indeed be the underlying cause of tolerance development. The initial research findings largely centred around transcriptional and translational changes in GABA_A_R subunit expression in various brain regions [30]. However, more recent studies report on structural changes in neuronal connectivity, including the loss of inhibitory GABAergic synapses [99] and dendritic spines [81, 103] in response to chronic diazepam treatments.

### Transcriptional and translational changes in GABA_A_R subunit expression

Changes in the relative subunit composition of GABA_A_Rs and a shift towards less sensitive receptor subtypes have been observed following the long-term benzodiazepine treatments [21]. Ultimately, if α and γ_2_ subunit expression is altered, clinical doses of benzodiazepines will be less effective requiring increased concentrations to maintain the same clinical outcomes. Given that different α subunits are linked to specific pharmacological effects of benzodiazepines and that tolerance to these effects develops at different rates, this hypothesis is alluring.

Indeed, numerous studies have demonstrated the subunit- and brain-region-specific changes in mRNA levels caused by chronic benzodiazepine treatments. Although the observed changes are inconsistent between the studies and do not directly correspond to changes at the protein level [104], downregulation of α_1_ [105–107] and γ_2_ subunit mRNA [108–112], and/or upregulation of benzodiazepine-insensitive subunits [30, 113, 114], have been implicated in the development of tolerance. Moreover, an increase in α_1_, β_3_ and γ_2_ expression together with redistribution of benzodiazepine-insensitive α_4_ subunits to synapses were observed following the induction of tolerance to sedative effects of diazepam in animal models [115]. A separate study reported that tolerance to sedative effects of diazepam involves prolonged activation of α_5_-GABA_A_Rs [116]. These inconsistent findings suggest that the impact of chronic benzodiazepines on subunit expression may vary by brain region, receptor subtypes and functional endpoint (e.g. sedation versus anxiolysis) and thus to what extent these changes contribute to the development of tolerance remains controversial. It is also important to note that benzodiazepines that target specific GABA_A_R subtypes are less likely to produce tolerance. For example, studies show that tolerance to α_1_-specific compound zolpidem does not readily develop in rodent models and in most clinical trials [30] where dosing frequency and clinical efficacy remain stable over 12–52 weeks of treatment [117]. However, tolerance and withdrawal have been observed in some case reports involving prolonged high dose use of this drug [118].

Thus, the interplay between different GABA_A_R subtypes in specific forms of tolerance may be one of the mechanisms involved, however further technological developments in animal and human studies are required to examine this hypothesis.

### Diazepam-dependent breakdown of inhibitory synapses

**Fig. 1:**
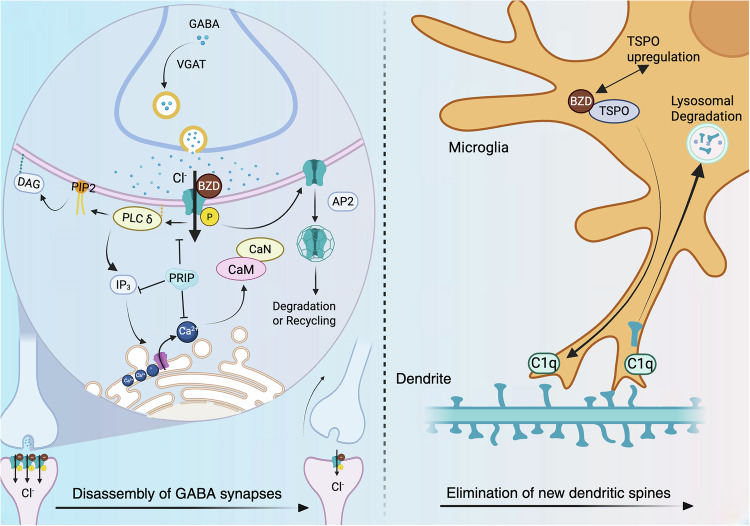
Impairments in inhibitory and excitatory synapses caused by chronic benzodiazepine treatments. *Left*. Benzodiazepine binding to GABA_A_Rs activates a metabotropic calcium signalling pathway mediated by PLCδ. PLCδ hydrolyses PIP_2_ into DAG and IP_3_. IP_3_ causes release of Ca^2+^ from ER stores and activation of calcineurin (CaN). CaN dephosphorylates the GABA_A_R to facilitate its interaction with AP2 and clathrin-mediated endocytosis. PRIP inhibits diazepam-induced endocytosis by competing with PLCδ for binding to GABA_A_Rs and by sequestering IP_3_ thereby inhibiting release of Ca^2+^ from the ER. Repeated activation of this pathway by benzodiazepines leads to depletion of synaptic pools of GABA_A_Rs and disassembly of GABAergic synapses. (Adapted from Nicholson et al., 2018). *Right*. At dendritic spines, chronic benzodiazepine exposure activates and upregulates microglial TSPO expression, triggering microglia-dependent elimination of newly formed spines through C1q deposition. However, pre-existing spines remain largely unaffected.

Beyond gene expression, long-term benzodiazepine exposure leads to structural degeneration of inhibitory synapses [99]. This was demonstrated by a significant reduction in inhibitory inputs to primary dendrites, a decrease in the size and number of postsynaptic γ_2_-GABA_A_R clusters and a lower frequency and amplitude of synaptic inhibitory currents (mIPSCs) measured in whole cell recordings. Detailed pharmacological characterization revealed that synaptic loss was driven by a metabotropic-like intracellular signalling pathway initiated upon diazepam binding and GABA activation of GABA_A_Rs (Fig. 1) [99]. This activation leads to dissociation of Phospholipase C δ (PLCδ) from the receptor and translocation to the plasma membrane, where PLCδ hydrolyses phosphatidylinositol 4,5-bisphosphate (PIP_2_) to generate diacylglycerol (DAG) and inositol trisphosphate (IP_3_). Subsequent binding of IP_3_ to its receptor on the smooth endoplasmic reticulum releases Ca^2+^ into the cytoplasm and activates the calcium/calmodulin-dependent phosphatase calcineurin which in turn dephosphorylates a specific serine residue 327 in the γ_2_ subunit (Ser327-γ_2_) and triggers endocytosis of GABA_A_Rs [99, 119]. Prolonged activation of this signalling pathway in cultured neurons in response to chronic diazepam caused a significant depletion of synaptic and intracellular GABA_A_R pools and subsequent disassembly of GABAergic synapses. Importantly, pharmacological blockade of specific enzymes involved in this pathway, including calcineurin, effectively blocked both processes. It is therefore tempting to speculate that cyclosporine or tacrolimus, calcineurin inhibitors already in clinical use as immunosuppressants [120], could be tested for their efficacy in alleviating benzodiazepine tolerance and dependence, although alternative administration routes and dosing may need to be considered to improve their safety profile. Importantly, this pathway was also blocked by a neuron-specific PLC-related but catalytically inactive protein 1 (PRIP1; [121]), which can outcompete PLCδ in binding to GABA_A_Rs. Given that PRIP1 is catalytically inactive, its binding to GABA_A_Rs may provide a temporary break on the PLCδ-driven synaptic loss and thus it may serve as a protective mechanism. In line with this is the phenotype of PRIP1/2 gene knockout mice, showing dysregulation of intracellular Ca^2+^ and calcineurin activity [122], decreased levels of synaptic γ_2_-GABA_A_Rs, reduced sensitivity to diazepam and increased anxiety-like behaviour [123].

This metabotropic signalling pathway may underlie not only receptor internalisation and synaptic loss, but also the long-term transcriptional changes required for the development of tolerance. Additionally, these inhibitory deficits may trigger adaptive changes in other neurotransmitter systems, including excitatory glutamatergic system, which has been implicated in the development of tolerance [30, 124]. The research described above provides a novel and convincing account of the mechanism underlying the development of benzodiazepine tolerance, and, although it was performed in vitro, it is consistent with the results of early in vivo studies [125] in which chronic benzodiazepine treatments were shown to reduce the extent of [^3^H]-flumazenil binding in the cortex, hypothalamus and hippocampus. While the approach has allowed detailed characterization of the signalling pathway activated by chronic diazepam treatments in neurons, further in vivo studies would be necessary to establish its causal relationship with the behavioural manifestations of benzodiazepines tolerance in preclinical models and patients.

## Long-term effects of benzodiazepines on excitatory synapses

**Fig. 2:**
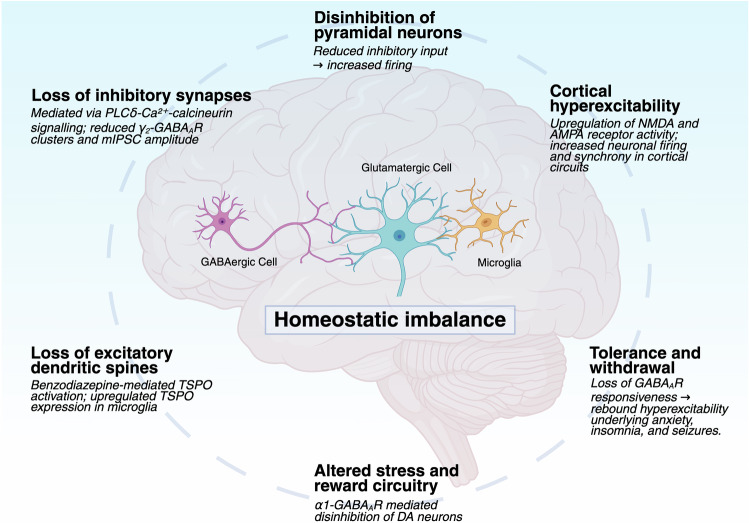
The Vicious Circle of Benzodiazepine Tolerance, Dependence, and Withdrawal. Chronic benzodiazepine exposure induces adaptive remodeling of GABAergic and glutamatergic synapses, disrupting excitatory–inhibitory balance and promoting cortical hyperexcitability, tolerance, dependence and withdrawal.

Long-term treatments of mice with diazepam have been shown to modify the structural organization of dendritic spines, the key postsynaptic components of glutamatergic excitatory synapses (Fig. 1) [81, 103]. Indicating a potential loss of glutamatergic synapses, these changes result primarily from the reduced formation and rapid elimination of new spines while the pre-existing spines remain largely unaffected. Interestingly, this diazepam-dependent loss of dendritic spines was not dependent on activation of GABA_A_Rs but instead on diazepam binding and upregulation of TSPO in the surrounding microglia [81]. This further led to deposition of complement component C1q on neuronal spines and their subsequent removal by microglia. Further pharmacological characterization using TSPO agonists and antagonists confirmed the key role of this protein in the observed pruning of dendritic spines [126]. Moreover, these changes correlate with changes in mouse behaviour that resemble cognitive decline seen in patients [127]. Although independent of diazepam-dependent loss of inhibitory synapses, these changes are likely to occur in parallel, contributing jointly to the long-lasting molecular, cellular and synaptic adaptations in the brain underpinning the development of benzodiazepine tolerance as schematically depicted in Fig. 2. Indeed, early in vivo studies largely support this hypothesis [30]. However, this remains controversial because of recently published observations that, instead of being weakened by chronic diazepam treatments, glutamatergic transmission is enhanced due to up-regulation of NMDA receptors and pro-excitatory signalling proteins [115]. In line with this study are earlier studies showing that benzodiazepine dependence could be reduced by co-administration of diazepam and NMDA-receptor antagonists [124] or genetic deletion of AMPA-receptor GluR-A subunits [128]. Thus, changes in glutamatergic synapses require further investigations to attest if strengthening of glutamatergic transmission could be possibly explained for example by increased incorporation of AMPA and NMDA receptors into the existing synapses in compensation for reduced structural plasticity and removal of new dendritic spines by microglia. Whether key to the development of benzodiazepine tolerance or not, changes in glutamate receptors and glutamatergic synapses provide a likely explanation for the robust withdrawal symptoms following an abrupt termination of chronic benzodiazepine use which are generally consistent with overexcitation in neuronal networks in animal models and in patients [129].

## Benzodiazepine tolerance in patients

As discussed above, it is well established that long-term use of benzodiazepines leads to profound changes in human brain physiology and behaviour, however it remains unknown whether and how these changes are underpinned by synaptic changes observed in animal models. Direct post-mortem evidence for synaptic or receptor adaptations specifically attributable to chronic benzodiazepine exposure and clinical tolerance has not been reported in the literature. Moreover, if such synaptic changes do indeed occur in the human brain, it would be important to define their tissue and brain region-specific localization, as well as whether they represent plastic changes that can be reversed upon drug cessation or permanent changes in synaptic structure, density and/or function. From the clinical perspective, the key question to address would be the critical time window during which these synaptic changes may become permanent and whether they can provide a biological explanation for a range of clinical symptoms associated with the development of tolerance.

Could the proposed synaptic changes underlying benzodiazepine tolerance resemble the changes observed in patients with benzodiazepine-resistant epilepsy? It is well documented that many patients exhibit resistance to the first-line antiepileptics, including diazepam, following prolonged episodes of status epilepticus [130] during which the hyperexcitability alters GABA_A_Rs in several aspects, including rapid downregulation of synaptic, benzodiazepine-sensitive α_1/2/3/5_, β_2/3_- and γ_2_-GABA_A_Rs [131–133], and upregulation of extrasynaptic, benzodiazepine-insensitive α_4_- and δ-GABA_A_Rs [132, 134, 135]. It is also important to note that the duration and frequency of seizures are good indicators of whether a patient will respond to a first-line benzodiazepine. Another potential mechanism underlying this resistance involves deficits in potassium/chloride cotransporter 2 (KCC2) function. This has been demonstrated in animal models of benzodiazepine-resistant seizures [136, 137], in which overexpression or activation of KCC2 increased the efficacy of diazepam in terminating seizures [138, 139]. These effects were linked to KCC2-dependent decrease in intracellular chloride and potentiation of hyperpolarizing effects of GABA_A_Rs. However, a possible role of KCC2 in benzodiazepine tolerance in seizure-naïve rodents and patients remains to be tested.

Similar changes in GABA_A_Rs were detected in patients who had multidrug-resistant temporal lobe epilepsy with recurrent seizures [140, 141], and in Positron Emission Tomography (PET) studies done with patients with refractory epilepsy [142, 143]. However, these receptor level alterations observed in benzodiazepine-resistant epilepsy may not be unique to seizure disorders. Related effects have been observed in patients with panic disorder, who exhibited reduced benzodiazepine receptor binding site availability in Positron Emission Tomography (PET) and single photon emission tomography (SPECT) studies [144, 145].

Overall, although neuroimaging techniques have been applied to investigate the effects of benzodiazepines, human studies directly addressing tolerance are scarce and largely confined to EEG assessments of acute tolerance rather than long-term adaptations [146, 147]. One notable exception is the study by Fujita et al. [148], in which changes in whole-brain GABA_A_R density measured by SPECT were correlated with sedation and memory impairment in patients in response to orally administrated alprazolam for 24 days [148]. GABA_A_R density was decreased within the first ten days of treatment but returned to baseline levels after three weeks of continuous dosing. Subjective sedation and memory impairment were evident from the first dose, with tolerance developing later (sedation by day 17 and memory by day 24). The authors concluded that transient downregulation of γ2-containing receptors contributes to but does not fully account for clinical tolerance. Limitations of this study include the small sample size and inability of SPECT to differentiate between synaptic and intracellular receptor pools. Nevertheless, this work provides the only human imaging evidence of receptor adaptations associated with benzodiazepine tolerance and suggests that synaptic adaptations precede measurable behavioural tolerance by approximately one week, supporting the hypothesis that synaptic changes are a driver of tolerance.

More recently, Brown et al. [149] conducted a longitudinal fMRI imaging in patients with generalised anxiety disorder receiving alprazolam in a standard twice-daily regimen. Clinically, significant anxiolytic effects emerged within the first week and were maintained after four weeks of continuous treatment. In terms of brain activity, the initial suppression of amygdala and anterior insula reactivity evident one hour after the first dose had dissipated by the four-week scan. Sedation, however, did not exhibit clear tolerance, as subjective sleepiness ratings remained elevated at the final visit. Exploratory whole-brain analyses revealed a late shift in activity towards fronto-striatal and parietal regions, suggesting that sustained symptom relief may depend on network reorganisation after the early limbic dampening has disappeared. Taken together, these results support a multi-phase model of benzodiazepine tolerance in which receptor-level adaptations are followed by broader circuit-level changes during sustained exposure. In a separate study by Wein et al. [150] the effects of prolonged alprazolam were analysed at high spatiotemporal resolution using resting-state fMRI in combination with behavioural testing following administration of a final dose, revealing changes in functional connectivity on a whole-brain level and within individual brain regions particularly in low activity level sensory regions which were correlated with sedation.

Although the key questions remain largely unanswered, recent methodological advancements offer new hope. These include correlative analyses of gene expression of inhibitory pre- and postsynaptic proteins and PET imaging with tracers selective for certain subtypes of GABA_A_R subtypes [151], development of new PET tracers with high specificity for particular GABA_A_R subtypes [152], ongoing work toward higher-resolution PET imaging in vivo detailing distinctive cortical and subcortical gradients of synapse density in the human brain [153], and neuroimaging biomarkers of early therapeutic responses in combination with machine learning and artificial intelligence that may predict the long-term effectiveness of targeted pharmacological interventions and preventive strategies [154]. Given the essential role of GABAergic synaptic transmission in many psychiatric diseases, the need for such integrative approaches at the brain region-, tissue-, cell- or synaptic level cannot be overstated.

## Benzodiazepine addiction

Benzodiazepines produce rewarding effects by facilitating dopamine transmission in the mesolimbic dopaminergic pathway in the brain [57]. These effects can be of a magnitude comparable to that of morphine at the doses tested, but they are mediated by different molecular mechanisms. Following the repeated exposure to benzodiazepines, dopaminergic neurons in the ventral tegmental area (VTA) show increased activity due to a decrease in their inhibition by GABAergic interneurons, a process termed disinhibition. This further leads to strengthening of glutamatergic synapses received by dopaminergic neurons due to an increase in the number of glutamate receptors. The increased activity of dopaminergic neurons leads to an increase in dopamine release in the limbic reward centres, including the nucleus accumbens [155]. The consequences of this were observed behaviourally in self-administration paradigms in mice and primates where a selective α_1_-GABA_A_R potentiation demonstrated enhanced reinforcing effects compared to α_2_-, α_3_-and α_5_-GABA_A_Rs [155, 156]. However, benzodiazepine-dependent structural changes in inhibitory synapses received by dopaminergic neurons remain to be studied. This would be an important line of future investigations because it may reveal additional benzodiazepine-activated changes in inhibitory synapses and neuronal connectivity contributing to the long-lasting disinhibition of dopaminergic neurons in VTA. The proposed mechanistic link between synaptic remodelling, persistent disinhibition of mesolimbic dopamine neurons, and compulsive use is largely consistent with clinical studies demonstrating that the continuous use of benzodiazepines for months and years in many patients is linked to severe withdrawal symptoms caused by physical dependence but also reward-associated effects of benzodiazepines [157, 158]. Could this diversity of behavioural responses in patients be linked to the dynamics of inhibitory synapse remodelling and elimination in affected brain regions as seen in animal models [159]? With the current development of new in vivo imaging technologies, this question may be possible to address experimentally in the foreseeable future.

## Conclusions

The first insights into the benzodiazepine-dependent changes in GABA_A_Rs dating back nearly four decades ago together with numerous in vitro and in vivo studies published over the years and the most recent studies shedding the light on their effects on neuronal connectivity demonstrate the enduring scientific pursuit towards better understanding of how the tolerance and dependence to these potent psychotropic drugs develop. The underlying molecular mechanisms proposed in the literature include both rapid and delayed adaptive changes, all of which are consistent with a reduction in the potentiating effects of benzodiazepines on GABA-induced chloride currents mediated by GABA_A_Rs. Within minutes to hours, benzodiazepines cause changes in GABA_A_Rs functional states, phosphorylation, protein-protein interactions, synaptic clustering and lateral mobility in and out of the synapse. Over more sustained periods of administration, benzodiazepines lead to altered subunit composition, synaptic depletion, breakdown of inhibitory synapses and disinhibition of neuronal circuits in parallel with microglia-mediated pruning of glutamatergic dendritic spines that may compromise excitatory plasticity and cognitive function. Although these changes may be interlinked or independent, direct evidence that any one of these processes contributes to benzodiazepine tolerance in patients is lacking. However, they offer a wide repertoire of neuronal proteins that could be genetically or pharmacologically manipulated to reach the level of knowledge required for the safer clinical use of benzodiazepines in patients. Further development of GABA_A_R subunit specific compounds, the targeting of PRIP, TSPO, neurosteroids, and glutamate plasticity to attenuate or reverse the acquisition of tolerance, as well as the development of high-resolution in vivo imaging and human in vitro models promise to bridge this translational gap. Exploring these avenues for research could produce a necessary paradigm shift in the management of anxiety, sleep and epilepsy disorders and the prescription of benzodiazepines. Seventy years after their discovery, moving beyond broad-spectrum benzodiazepines towards synapse-informed, subtype-tailored interventions tackling benzodiazepine tolerance is not only a pharmacological challenge, but a necessary step to improve care for patients living with anxiety, epilepsy and related neuropsychiatric disorders.
